# RETRACTION: Spliceosomal Protein E Regulates Neoplastic Cell Growth by Modulating Expression of Cyclin E/CDK2 and G2/M Checkpoint Proteins

**DOI:** 10.1111/jcmm.71228

**Published:** 2026-06-01

## Abstract

**RETRACTION**: LiZ. and PützerB. M. , "Spliceosomal Protein E Regulates Neoplastic Cell Growth by Modulating Expression of Cyclin E/CDK2 and G2/M Checkpoint Proteins," Journal of Cellular and Molecular Medicine 12, no. 6a (2008): 2427‐2438. 10.1111/j.1582-4934.2008.00244.x.18208561 PMC4514120

The above article, published online on 16 December 2008 in Wiley Online Library (wileyonlinelibrary.com), has been retracted by agreement between the journal Editor‐in‐Chief, Stefan Constantinescu; and John Wiley & Sons Ltd. A third party reported that comments had been made on PubPeer [1] that raised concerns about potential duplication of bands in Figure 2B. Additional investigation by the publisher found evidence of splicing in Figure 1B and duplication of Actin blots between Ad‐GFP in Figure 3A and Ad‐Sme pre‐mRNA splicing in Figure 5A. The investigation also found evidence of duplications of the Ad SmE Actin blot in Figure 3A and the Ad‐GFP p‐CDC2 (Tyr15)/CDC2 blot in Figure 5A. Additional potential duplicated bands were detected in Figure 5A.

The authors responded to an inquiry by the publisher and stated that the Western blots were generated separately for each experiment and were not intentionally duplicated or altered. However, the original data were no longer available due to the time that had elapsed since publication.

The retraction has been agreed to because the evidence of highly similar or duplicated bands fundamentally compromises the editors’ confidence in the results presented. The authors disagree with the retraction.

References

[1] Unregistered Submission and Hydroporus ferrugineus, Comments on “Spliceosomal protein E regulates neoplastic cell growth by modulating expression of cyclin E/CDK2 and G2/M checkpoint proteins,” PubPeer, May 2016‐October 2025. https://pubpeer.com/publications/216D30610A95CE3D109B97FC1CA64D

**RETRACTION**: Z. Li and B. M. Pützer , "Spliceosomal Protein E Regulates Neoplastic Cell Growth by Modulating Expression of Cyclin E/CDK2 and G2/M Checkpoint Proteins," Journal of Cellular and Molecular Medicine 12, no. 6a (2008): 2427‐2438. 10.1111/j.1582-4934.2008.00244.x.18208561 PMC4514120

The above article, published online on 16 December 2008 in Wiley Online Library (wileyonlinelibrary.com), has been retracted by agreement between the journal Editor‐in‐Chief, Stefan Constantinescu; and John Wiley & Sons Ltd. A third party reported that comments had been made on PubPeer [1] that raised concerns about potential duplication of bands in Figure 2B. Additional investigation by the publisher found evidence of splicing in Figure 1B and duplication of Actin blots between Ad‐GFP in Figure 3A and Ad‐Sme pre‐mRNA splicing in Figure 5A. The investigation also found evidence of duplications of the Ad SmE Actin blot in Figure 3A and the Ad‐GFP p‐CDC2 (Tyr15)/CDC2 blot in Figure 5A. Additional potential duplicated bands were detected in Figure 5A.

The authors responded to an inquiry by the publisher and stated that the Western blots were generated separately for each experiment and were not intentionally duplicated or altered. However, the original data were no longer available due to the time that had elapsed since publication.

The retraction has been agreed to because the evidence of highly similar or duplicated bands fundamentally compromises the editors’ confidence in the results presented. The authors disagree with the retraction.

**References**

[1] Unregistered Submission and Hydroporus ferrugineus, Comments on “Spliceosomal protein E regulates neoplastic cell growth by modulating expression of cyclin E/CDK2 and G2/M checkpoint proteins,” PubPeer, May 2016‐October 2025. https://pubpeer.com/publications/216D30610A95CE3D109B97FC1CA64D

